# Contribution of DECT in detecting serosal invasion of gastric cancer

**DOI:** 10.3906/sag-1811-168

**Published:** 2019-06-18

**Authors:** Ali KÜPELİ, Eser BULUT, Ayşegül CANSU, Ali GÜNER, Mehmet SOYTÜRK, Gürkan DANIŞAN

**Affiliations:** 1 Department of Radiology, Faculty of Medicine, Erzincan Binali Yıldırım University, Erzincan Turkey; 2 Department of Radiology, Trabzon Kanuni Training and Research Hospital, Trabzon Turkey; 3 Department of Radiology, Faculty of Medicine, Karadeniz Technical University, Trabzon Turkey; 4 Department of General Surgery, Faculty of Medicine, Karadeniz Technical University, Trabzon Turkey5; 5 Department of Radiology, Muş State Hospital, Muş Turkey

**Keywords:** DECT, gastric cancer, iodine concentration

## Abstract

**Background/aim:**

This study aimed to investigate the relationship between the iodine concentration (IC) of perigastric fat tissue as assessed by dual-energy computed tomography (DECT) and serosal invasion of gastric cancer.

**Materials and methods:**

A total of 41 patients underwent preoperative staging evaluation for gastric cancer using DECT between July 2015 and March 2018. Patients were divided into 2 groups based on pathology results: serosal invasion (stage T4a) and intact serosa (stages T1–T3). Cutoff values, the diagnostic efficacy of IC in the perigastric fat tissue, and the perigastric fat tissue/tumor (P/T) ratio were determined.

**Results:**

Among the 41 patients, 22 had stage T4a gastric cancer and 19 patients had gastric cancer with a stage lower than T4a. The mean IC of perigastric fat tissue and the P/T ratio were significantly higher in patients with serosal invasion than in those with intact serosa (P < 0.001). During the arterial phase, the area under the curve (AUC) was 0.915 and 0.854 for the IC of perigastric fat tissue and the P/T ratio, respectively. During the venous phase, the AUC was 0.890 and 0.876 for the IC of perigastric fat tissue and the P/T ratio, respectively.

**Conclusion:**

The IC in the perigastric fat tissue seems to be a reliable indicator for serosal invasion of gastric cancer.

## 1. Introduction

Gastric cancer remains one of the most important causes of cancer-related deaths worldwide (1–3). Early, correct diagnosis and precise preoperative staging of gastric cancer are very important because these patients can be treated with laparoscopic or minimally invasive surgery (4). The 5-year survival rate in patients with early gastric cancer is 85%–100%, whereas that in patients with advanced gastric cancer is 7%–27% (4). The TNM staging system is usually used to stage gastric cancer, and T4 is described as a tumor that has spread through all the muscle layers outside the stomach and invades the serosa or adjacent structures (3). Selection of the appropriate treatment options, including neoadjuvant chemotherapy or multiorgan surgery, depends on correct differentiation between stage T4 gastric cancer and T3 or earlier stages (5–7).

Computed tomography (CT) is the most common method used to identify the stages of gastric cancer and assess the local extension of the tumor, nodal disease, and metastases with up to 80%–89% accuracy (8). Moreover, magnetic resonance imaging, endoscopic ultrasonography, and positron emission tomography can be used in tumor staging.

Dual-energy CT (DECT) is a newly developed technology, with the main principle based on the application of 2 distinct energy settings that can be used to distinguish materials composed of different molecular compounds according to their attenuation characteristics (9,10). DECT can provide material-specific imaging instead of attenuation-based imaging. For example, the presence of iodine, calcium, barium, and uric acid in an image can be obtained with a single examination (9,10). Additionally, the presence and amount of the targeted material, such as iodine, can be evaluated in a lesion or an organ. After DECT images are obtained, multiple datasets, such as virtually unenhanced images, monochromatic images, elemental decomposition analyses, and iodinated attenuation maps can be acquired simultaneously (11).

Accurate material decomposition from DECT images can be used to obtain the quantitative iodine concentration (IC). DECT can contribute to tumor detection, lesion characterization, and evaluation of the response to neoadjuvant chemotherapy in oncology patients. A phantom study showed that the IC calculated from DECT images indicates the actual IC (12). Furthermore, the IC can be used as an imaging biomarker to estimate the efficacy of neoadjuvant chemotherapy in patients with gastric cancer (13,14).

Therefore, the IC measured from the perigastric adipose tissue of the lesser and greater omentum may be used to evaluate the stage of tumor invasion in patients with gastric cancer. In this study, we aimed to investigate the relationship between the IC of perigastric fat tissue and serosal invasion of gastric cancer with histopathological results as a reference.

## 2. Materials and methods

### 2.1. Study population

Between July 2015 and March 2018, 45 patients were evaluated with preoperative CT and DECT to determine the stage of gastric cancer, which was confirmed by endoscopic biopsy. Four patients were excluded from the study due to an allergy to the contrast medium (n = 1), oral contrast agent administration (n = 1), or known stage T4b cancer (n = 2). Therefore, 41 patients were included in the study. A final diagnosis was obtained based on the histopathologic analysis of resected specimens after surgery. The patients’ CT images were reevaluated by 2 radiologists who were blinded to the endoscopic findings and pathological results until a consensus was reached. The patients’ demographic characteristics were recorded. Patients were divided into 2 groups based on postoperative pathological results: serosal invasion (stage T4a) and intact serosa (stages T1–T3). The study was approved by the institutional ethics committee, and written informed consent was obtained from all patients before DECT examination.

### 2.2. CT protocol

All CT examinations were performed using a 160-slice dual-energy CT scanner (Toshiba Aquilion; Toshiba Medical Systems, Otawara, Japan). All patients fasted for at least 8 h before the CT examination and drank 500–1000 mL of water to expand the stomach just before CT examination. DECT images were obtained at 25 s and 70 s after administration of nonionic contrast agent (arterial phase and portal venous phase, respectively) to each patient based on their weight (2 mL/kg) and 30 mL of saline injection at a flow rate of 4 mL/s. The arterial and portal venous phase images were obtained using dual-energy scan mode with a tube voltage of 80 kVp and 130 kVp with a tin filter, a tube current of 230 and 150 mAs, a collimation of 32 × 0.6 mm for both tubes, a pitch of 0.55, and a gantry rotation time of 0.5 s. The images were reconstructed with a 1.5-mm thickness. Moreover, the images were reconstructed with a 5-mm slice thickness to measure the IC during the arterial and portal venous phases.

### 2.3. Analysis of CT and DECT images

**Figure 1:**
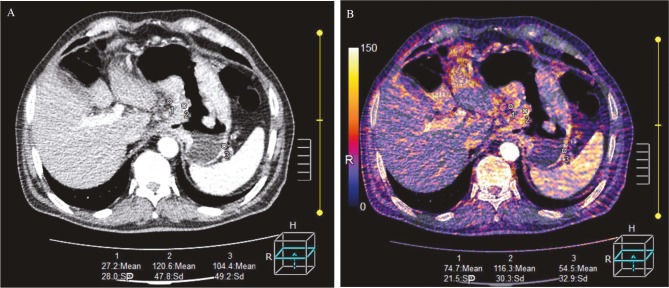
Representative CT images obtained from a 58-year-old female patient with adenocarcinoma with invasion of serosa. (A) The mixed venous phase image shows thickening of the wall of the antrum with transmural enhancement. (B) The iodine shows that the iodine concentrations were 74.7 mg/mL in the perigastric fat (ROI 1), 116.3 mg/mL in the tumor (ROI 2), and 54.5 mg/mL in the normal gastric wall (ROI 3).

Monochromatic arterial and venous phase images were acquired by combining high- and low-energy images at a 1:1 ratio. These images were considered as simulated single-energy 120-kVp images for analyzing the diagnostic performance of CT to detect serosal invasion of gastric cancer. Image analysis and measurements were reevaluated using the standard dual-energy software on the CT console (Version 6, Toshiba Medical Systems). The arterial and venous phase images derived from high- and low-energy images were both reconstructed with 5-mm slice thickness. First, the region of interest (ROI) was placed on the normal gastric wall and the tumor tissue, and then the ROI was positioned on perigastric fat tissue close to the tumor to measure the IC in the involved gastric serosa (Figure 1). A circular or elliptical ROI measuring 25–50 mm2 (avoiding vascular structures) was used. All measurements were performed at least 3 times, and the average values were recorded. Moreover, the ICs during the arterial and venous phases were calculated repeatedly. An ROI of the same size and shape was placed at the same anatomic location.

### 2.4. Statistical analysis

All of the data were analyzed using SPSS 13.0 (SPSS Inc., Chicago, IL, USA) and the MedCalc package (MedCalc Statistical Software version 16.8, MedCalc Software bvba, Ostend, Belgium). The means and ranges of age and the ICs of the tumor, perigastric fat tissue, and normal gastric wall were calculated. Additionally, the IC of the perigastric fat tissue/tumor ratio (P/T) was noted during the arterial and portal venous phases. The Kolmogorov–Smirnov test was used to evaluate deviation from the normal distribution. The parametric Student t-test was used to compare the ICs of the tumor, perigastric fat tissue, and normal gastric wall. The optimal cutoff points for the ICs for the determination of serosal invasion of gastric cancer were found using receiver operating characteristic (ROC) curve analysis. If the obtained IC was less than the given cutoff value, the patient was considered to have no serosal invasion. If the obtained IC was greater than the given cutoff value, the patient was considered to have serosal invasion. The sensitivity, specificity, positive predictive value (PPV), negative predictive value (NPV), and accuracy of these parameters were calculated. Furthermore, the diagnostic performance of the CT evaluation was noted. The area under the fitted binormal ROC curve (AUC) was used to measure the diagnostic efficacy. The AUC values were calculated and compared using these parameters. P < 0.05 was considered to indicate significant difference.

## 3. Results

In the present study, 41 patients (31 male, 10 female; age range: 30–89 years; mean age: 63 years) underwent preoperative abdominal DECT for gastric cancer staging. According to the postoperative pathology results, 19 patients had gastric cancer of less than stage T4a with intact serosa, and 22 patients had stage T4a gastric cancer with serosal invasion. The histopathologic diagnoses were 13 well-differentiated adenocarcinomas, 15 moderately differentiated adenocarcinomas, 7 poorly differentiated adenocarcinomas, 5 mucinous adenocarcinomas, and 1 signet ring cell carcinoma. The locations of the gastric tumors were the antrum in 13 patients, the lesser curvature in 11 patients, the greater curvature in 8 patients, the cardia in 5 patients, and the corpus in 4 patients.

Based on images obtained using conventional CT, 17 of 19 patients were classified as having gastric cancer of less than stage T4a with intact serosa, and 16 of 22 patients were correctly diagnosed with stage T4a gastric cancer during arterial phase imaging. Moreover, 17 of 19 patients were classified as having gastric cancer below stage T4a with intact serosa, and 17 of 22 patients were correctly diagnosed with stage T4a gastric cancer during venous phase imaging. The accuracies of conventional CT were 0.809 and 0.829 during the arterial and venous phases, respectively.

**Figure 2:**
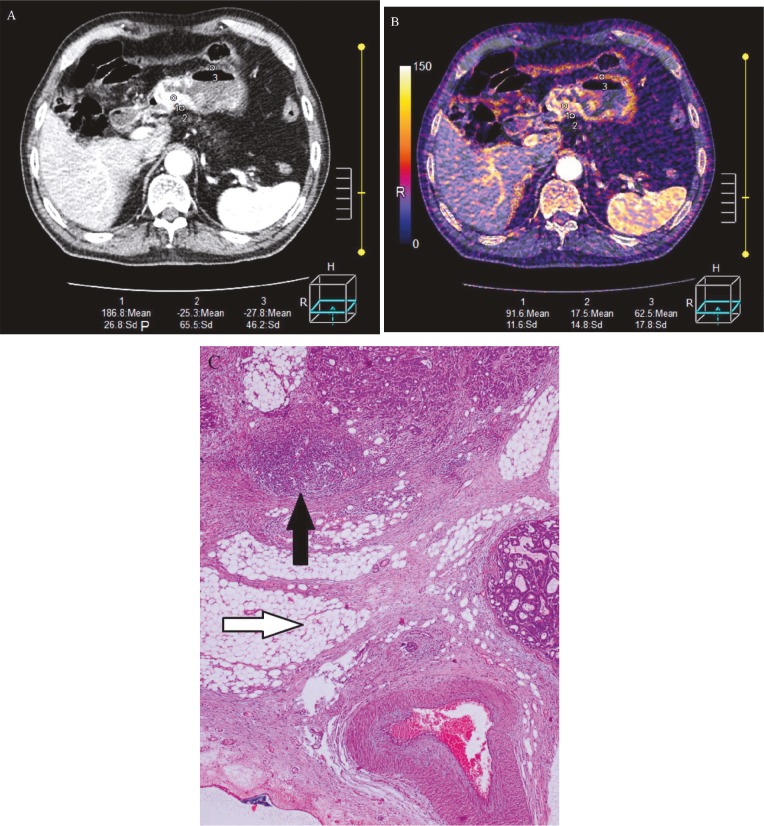
Representative CT images obtained from a 41-year-old male patient with adenocarcinoma without invasion of serosa with transmural enhancement. (A) The mixed arterial phase image shows thickening of the wall of the antrum. (B) The iodine shows that the iodine concentrations were 17.5 mg/mL in the perigastric fat (ROI 1), 91.6 mg/mL in the tumor (ROI 2), and 62.5 mg/mL in the normal gastric wall (ROI 3). (C) The histological image with hematoxylin and eosin (HE, 100×) shows stage T3 adenocarcinoma (black arrow) without invasion of surrounding submucosa (white arrow).

Table 1 shows the mean ICs of the tumor, perigastric fat tissue, and normal gastric wall and the P/T ratio. During the arterial phase, the mean ICs of the tumor, perigastric fat tissue, and normal gastric wall and the P/T ratio were 75.8 mg/mL, 57.0 mg/mL, 45.4 mg/mL, and 0.83, respectively. During the portal venous phase, the mean ICs of the tumor, perigastric fat tissue, and normal gastric wall and the P/T ratio were 92.1 mg/mL, 66.2 mg/mL, 54.4 mg/mL, and 0.77, respectively. The mean IC in the perigastric fat tissue and the T/P ratio were significantly higher in patients with serosal invasion than in those without serosal invasion (P < 0.001) (Figure 2). However, the differences in the mean ICs of the tumor and the normal gastric wall between patients with and those without serosal invasion were not significant.

**Table 1:** Patient characteristics.

	Total	Serosal invasion		P-value	Present	Absent
		Number	41		22	19	
Arterial phase				
IC values of tumor (mg/mL)	75.8 ± 23.7	73.1 ± 24.3	78.8 ± 23.2	0.445
Normal gastric wall (mg/mL)	45.4 ± 15.9	45.7 ± 15.9	45.0± 16.4	0.917
IC values of PFT (mg/mL)	57.0 ± 17.3	68.3 ± 13.0	44.0 ± 11.6	<0.001
P/T ratio	0.83 ± 0.4	1.04 ± 0.42	0.59 ± 0.18	<0.001
Venous phase				
IC values of tumor (mg/mL)	92.1 ± 28.8	88.7 ± 27.9	96.1 ± 23.2	0.425
Normal gastric wall (mg/mL)	54.4± 16.6	54.5± 18.6	54.4 ± 14.4	0.875
IC values of PFT (mg/mL)	66.2 ± 21.1	79.1 ± 17.2	51.4 ± 14.1	<0.001
P/T ratio	0.77 ± 0.3	0.95 ± 0.29	0.57 ± 0.18	<0.001

PFT: Perigastric fat tissue; P/T: perigastric fat tissue/tumor.

**Figure 3:**
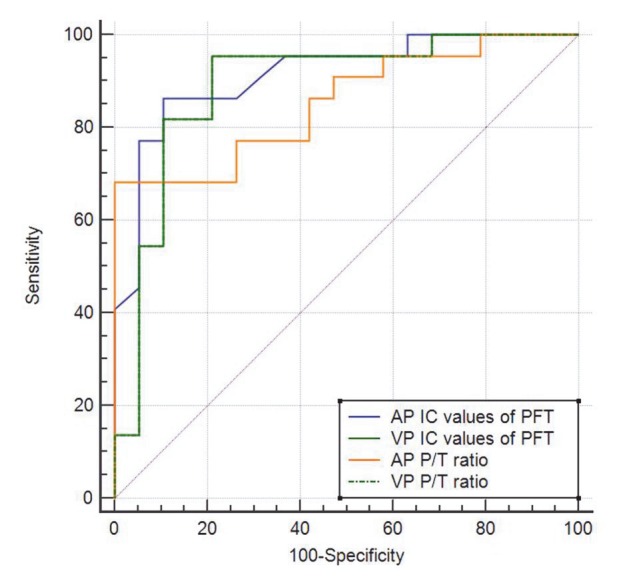
The graph shows the receiver operating characteristic curve for the iodine concentration (IC) values of perigastric fat tissue (PFT) and perigastric fat tissue/tumor (P/T) ratio in the arterial phase (AP) and venous phase (VP).

For arterial and portal venous phase imaging, the ROC curves for the IC of the perigastric fat tissue and the P/T ratio are shown in Figure 3. The AUCs were 0.915 and 0.854 for the IC of the perigastric fat tissue and the P/T ratio, respectively. The optimal cutoff values obtained from the ROC curve analysis that provided the highest sensitivity and specificity for the IC of the perigastric fat tissue and the P/T ratio were 56 mg/mL and 0.71, respectively. The diagnostic values obtained with these cutoff values are presented in Table 2. The AUCs were 0.890 and 0.876 for the IC of the perigastric fat tissue and the P/T ratio, respectively. The optimal cutoff values obtained from the ROC analysis that provided the highest sensitivity and specificity for the IC of the perigastric fat tissue and the P/T ratio were 60 mg/mL and 0.68, respectively. The AUCs for the IC of the perigastric fat tissue and the T/P ratio did not show significant differences during the arterial and portal venous phases (P > 0.692).

**Table 2:** Results of receiver operating characteristic (ROC) analysis for IC values of PFT and P/T ratio.

	AUC	Cutoff Level	Sensitivity (%)	Specificity (%)	PPV (%)	NPV (%)	Accuracy (%)
Arterial phase							
IC values of PFT	0.915	56 (mg/mL)	86.3	89.4	90.5	85.0	87.8
P/T ratio	0.854	0.71	77.2	73.6	77.3	73.7	73.1
Venous phase							
IC values of PFT	0.890	60 (mg/mL)	81.8	89.4	90.0	81.0	85.3
P/T ratio	0.876	0.68	81.8	78.9	81.8	78.9	80.4

AUC: Area under the curve; PPV: positive predictive value; NPV: negative predictive value; PFT: perigastric fat tissue; P/T: perigastric fat tissue/tumor.

## 4. Discussion

In this present prospective study, we investigated the role of the IC measured from perigastric adipose tissue for evaluating serosal invasion of gastric cancer. Our results showed that the IC of the perigastric fat tissue and the P/T ratio could be used to evaluate serosal invasion of gastric cancer during the arterial and portal venous phases. The IC of the perigastric fat tissue and the P/T ratio had 87.8% and 73.1% accuracy, respectively, during the arterial phase and 85.3% and 80.5% accuracy, respectively, during the portal venous phase for determining serosal invasion of gastric cancer.

Clinically, DECT has improved tumor detection and staging by reducing the scan time and radiation dose during oncological examination. Iodine measurements made with DECT are a quantitative imaging method for the advanced assessment of tumors with iodine uptake (15). With the increasing clinical use of DECT, this imaging modality has been implemented for various whole-body routine oncological monitoring, as in gastric cancer (16). In this study, patients with serosal invasion of gastric cancer had a significantly higher IC in the perigastric fat tissue adjacent to the tumor during both the arterial and portal venous phases than those without tumor invasion. Additionally, Yang et al. showed that ICs were significantly higher in patients with serosal invasion due to gastric cancer than in those without serosal invasion (16). When the tumor spreads to the serosa, stromal reactions, such as increased fibroblasts, inflammatory and immune cells, proliferating vessels, and noncellular matrix components, play important roles in cancer progression (17). The higher IC in the perigastric fat tissue of patients with serosal invasion is probably related to increased perfusion, possibly due to proliferating vessels induced by tumor invasion or leakage from malignant cell membranes.

Chemotherapy or chemoradiotherapy can be performed preoperatively in patients with resectable tumors or those with nodal metastasis. On CT images, increased density of fat tissues at the serosal surface is commonly used to define serosal invasion of gastric cancer, whereas reactive fibrous connective tissue hyperplasia in the serosa may also lead to increased density (16). Although thin-slice CT images, multiplanar reformation techniques, and virtual gastroscopy can effectively assess the extent of gastric cancer, the specificity of CT imaging for detecting serosal invasion remains relatively low (18–21). In our study, 3 and 4 patients had stage T4a lesions diagnosed during the arterial and venous phases, respectively, that were underestimated as lesions that were below stage T4a. These findings show the limitations of using CT for detecting serosal invasion of gastric cancer.

The accuracy of CT in staging gastric cancer has been reported to be approximately 80%–89% (8,18–21). Consistent with previous studies, we obtained approximately 80% accuracy of CT for detecting serosal invasion of gastric cancer. Additionally, this study revealed that ICs obtained with DECT may be promising for defining stage T4a gastric cancer with diagnostic performance similar to that of multiplanar images. However, IC still cannot be used to detect serosal invasion in all patients. In our study, 19 of 22 patients with serosal invasion were correctly classified using DECT. Approximately 80% sensitivity and 85% specificity were found during the arterial and venous phases; however, ICs alone were not suitable for differentiating these patients. ICs as calculated by DECT can contribute to the use of single-energy CT for the diagnosis of stage T4a gastric cancer.

DECT provides both quantitative and qualitative data. Iodine maps are color maps; better color distinction can be obtained with iodine maps than with grayscale images obtained with a conventional multidetector CT scanner. Additionally, ICs obtained from DECT are numerical data; thus, objective diagnosis and evaluation can be performed. Increased perfusion due to abnormal tumor angiogenesis occurs in areas invaded by cancer cells. The IC in the serosal fat tissue could be used to evaluate increased perfusion. Therefore, ICs obtained with DECT may show more objective and accurate diagnostic performance for detecting serosal invasion of gastric cancer than other parameters. Furthermore, DECT can facilitate detection of tumor invasion of adjacent vascular structures with the use of monochromatic images (22,23).

This study has a number of limitations. First, we did not evaluate the inter- or intraobserver variability in the study. The second limitation is that there were not enough patients to investigate the effectiveness of ICs for differentiating subtypes of gastric cancer. The third limitation is that normalized iodine values were not evaluated. The fourth limitation is that benign and malignant lymph node staging was not performed.

In conclusion, the IC measured in the perigastric fat tissue seems to be a reliable technique for detecting serosal invasion of gastric cancer. However, extensive studies with larger populations are needed to clearly confirm the effectiveness of this method.
